# Editorial: Advancements in proteomics and PTMomics: unveiling mechanistic insights and targeted therapies for metabolic diseases

**DOI:** 10.3389/fcell.2025.1556129

**Published:** 2025-01-24

**Authors:** Xiulan Chen, Yanchang Li, Daniela Braconi

**Affiliations:** ^1^ Laboratory of Proteomics, Institute of Biophysics, Chinese Academy of Sciences, Beijing, China; ^2^ State Key Laboratory of Medical Proteomics, Beijing Proteome Research Center, National Center for Protein Sciences (Beijing), Institute of Lifeomics, Beijing, China; ^3^ Department of Biotechnology, Chemistry, and Pharmacy, University of Siena, Siena, Italy

**Keywords:** proteomics, PTMomics, metabolic diseases, proteoform, cellular pathology

The growing prevalence of metabolic diseases (including type 2 diabetes, hypertension, hyperlipidemia, obesity, and non-alcoholic fatty liver disease) poses a significant economic burden to our society (Chew et al., 2023). The pathogenesis of metabolic diseases is a multifactorial pathophysiological process arising from various systematic metabolic defects; therefore, a comprehensive, holistic approach is needed to deeply investigate the mechanisms associated with the development of metabolic diseases.

In the last 2 decades, proteomics, the large-scale study of proteins, has emerged as a powerful and useful tool to investigate the roles of proteins in biological systems. The rapid development of MS technologies has enabled a system-wide, qualitative, and quantitative analysis of the proteome at the cell, tissue, or organism level (Martinez-Val et al., 2022).

Proteomic studies can be classified in three main categories, i.e., expression, functional and structural proteomics. Expression proteomics involves the qualitative and quantitative comparison of protein expression in the entire proteome of samples from different states. Different MS-based quantitative proteomic techniques, such as SILAC, dimethyl labeling, iTRAQ, TMT, label-free, and DIA, have been applied to highlight differentially expressed proteins in different states, such as health and disease states. However, these MS-based quantitation methods are characterized by different features, advantages, and shortcomings (Chen et al., 2021). The choice of suitable quantitative methods is necessary for accurate and reliable results.

Post-translational modifications (PTMs) of proteins, the covalent modifications involving the addition or removal of chemical/protein groups on proteins, are highly important for the regulation of protein function, localization, interaction, and activity both in physiological and disease states. MS technologies were implemented as important tools to characterize and discover novel PTMs (Wang et al., 2019). PTMomics, which is the MS-based qualitative and quantitative analysis of PTMs in a given organism or cell, holds great potential to elucidate disease mechanisms by providing knowledge on the nature and regulation of PTMs, contributing significantly to structural and functional proteomic studies. However, the MS-based analysis of PTMs poses significant technical challenges due to the low-abundance and lability of PTM-modified peptides. Development of sensitive PTM enrichment methods could facilitate the identification and quantification of PTMs in complex samples. In the work by Ye et al., high-affinity antibody enrichment combined with high-resolution LC-MS/MS is used to systematically investigate acidic lysine acylations (malonylation, succinylation, and glutarylation) in *Mycobacterium smegmatis*, with protein-protein interaction networks and pathway enrichment analysis suggesting a complex mechanism through which Mycobacteria might adapt to the host cellular environment manipulating the host’s metabolic environment. Interestingly, these authors imply the potential utility of acidic acylations as biomarkers or therapeutic targets.

Understanding the complexity of proteoforms, i.e., protein variants generated by mutations, alternate splicing, mRNA processing and PTMs, is crucial for the selection of biomarkers, therapeutic targets and drug candidates. By using commercial, highly purified serum albumin as a model, Woodland et al. show how the most common current analytical MS approaches (shotgun or bottom-up proteomics and mass spectrometry-intensive top-down proteomics) both fail to fully and effectively identify proteoforms and/or provide their comprehensive analysis, proposing high-resolution, quantitative integrated/integrative top-down proteomics as a better approach while raising interesting questions in readers’ minds.

To circumvent analytical challenges, several computational approaches have been developed to study PTMs, such as phosphorylation, glycosylation, S-nitrosylation, methylation, sumoylation, palmitoylation, and N-myristoylation (Audagnotto and Dal Peraro, 2017). These *in silico* bioinformatics tools could predict modified sites that can be then validated with experimental approaches, expanding the scope of PTM studies. In this research topic, Zhang et al. applied deep learning combined with attention mechanism to develop a tool called DeepO-GlcNAc for prediction of protein O-GlcNAcylation. It showed that DeepO-GlcNAc predictor achieved remarkable performance in prediction of O-GlcNAc sites with an accuracy of 92% and an average precision of 72%. This DeepO-GlcNAc predictor is a valuable tool for future research in protein O-GlcNAcylation.

Gastrointestinal dysfunctions are often associated with type 2 diabetes mellitus (T2DM), a complicated metabolic illness. Since pathophysiology remains unknown, it is critically important to investigate risk factors and preventative strategies for gastrointestinal disorders linked to T2DM. Zhang et al. performed a comprehensive analysis of the gastric sinus metabolome, transcriptome, and proteome in db/db mice to explore the possible causes behind gastrointestinal dysfunctions caused by T2DM. The authors used multi-omics research to reveal and prove that genes, proteins, and metabolites in the T2DM-induced gastroenteropathy mice group were involved in arachidonic acid metabolism, glycerophospholipid metabolism and vitamin digestion and absorption, which would provide vital understandings of the pathophysiology.

Given the complexity of biological systems, single omics analyses cannot provide a comprehensive understanding of molecular changes of complex diseases. Multi-omics, which combines two or more omics, such as genomics, transcriptomics, proteomics, metabolomics, could provide an integrated approach for deeper insights and discoveries and promote the understanding of human diseases (Chen et al., 2020).
